# Protective Effect of MFG-E8 on Necroptosis-Induced Intestinal Inflammation and Enteroendocrine Cell Function in Diabetes

**DOI:** 10.3390/nu14030604

**Published:** 2022-01-29

**Authors:** Xiaomin Hua, Baoying Li, Fei Yu, Wenqian Zhao, Yuwei Tan, Xiaoli Li, Chunli Fu, Xin Yu, Haiqing Gao, Mei Cheng

**Affiliations:** 1 Department of Geriatric Medicine, Qilu Hospital, Cheeloo College of Medicine, Shandong University, Jinan 250012, China; huaxiaomin666@sina.com (X.H.); feifeifei18@163.com (F.Y.); abigailzhongxiyi@163.com (W.Z.); 18189177395@163.com (Y.T.); ffcl2001@163.com (C.F.); yuxindoctor@163.com (X.Y.); 13573187788@163.com (H.G.); 2 Key Laboratory of Cardiovascular Proteomics of Shandong Province, Qilu Hospital, Cheeloo College of Medicine, Shandong University, Jinan 250012, China; libaoying77@163.com; 3 Department of Geriatric Medicine, Qilu Hospital (Qingdao), Cheeloo College of Medicine, Shandong University, Qingdao 266035, China; 4 Jinan Aixinzhuoer Medical Laboratory, Jinan 250100, China; 5 Department of Pharmacy, Qilu Hospital, Cheeloo College of Medicine, Shandong University, Jinan 250012, China; lixiaoli7576@163.com

**Keywords:** milk fat globule epidermal growth factor 8, advanced glycation end products, necroptosis, intestinal inflammation, D-pinitol

## Abstract

Low-grade inflammation is one of the characteristics of metabolic disorders induced by diabetes mellitus. The present study explores the underlying mechanism of milk fat globule epidermal growth factor 8 (MFG-E8) on necroptosis-induced intestinal inflammation and intestinal epithelial endocrine cell dysfunction in diabetes. Compared with the normal control group, pathological changes such as blunt and shortened villus and denuded villus tips were observed in ileum tissue of streptozotocin (STZ) induced senescence-resistant 1 (SAMR1) and senescence-accelerated prone 8 (SAMP8) diabetic mice under light microscope. Western blotting and immunohistochemistry (IHC) displayed significantly decreased protein expression of MFG-E8 in SAMR1 and SAMP8 diabetic mice, accompanied by an increased expression of phosphorylated mixed lineage kinase domain-like (p-MLKL) and HMGB1. In addition, advanced glycation end products (AGEs) significantly increased the pro-inflammatory mediators (TNF-α, IL-1β, IL-6) and HMGB1 by activating the receptor-interacting protein kinase 3 (RIPK3)/MLKL signaling pathway in enteroendocrine STC-1 cells. D-pinitol pretreatment markedly attenuated the release of pro-inflammatory mediators and increased the expression of MFG-E8. MFG-E8 small interfering RNA (siRNA) promoted, while MFG-E8 overexpression inhibited, the activation of receptor-interacting proteins (RIPs) pathway and pro-inflammatory factors. Our study demonstrated that downregulation of MFG-E8 is an important phenomenon in the pathogenesis of diabetes-related intestinal inflammatory damage. MFG-E8 overexpression and D-pinitol intervention could protect against necroptosis-induced intestinal inflammation and maintain the function of enteroendocrine STC-1 cells in diabetes.

## 1. Introduction

Type 2 diabetes is thought to be caused by multiple risk factors such as age, obesity, or an unhealthy lifestyle. High fat or high sugar diet can cause low-grade inflammation and change the composition of intestinal microbiota, so as to promote metabolic endotoxemia and intestinal epithelial damage [1]. The accumulation of advanced glycation end products (AGEs), as an important mechanism in the pathogenesis of diabetes, can cause oxidative stress damage and lead to cell dysfunction or even death, which eventually leads to multiple histiocytic damage in diabetes [2] When enteroendocrine cells distributed in the intestinal epithelium are continuously exposed to high concentrations of AGEs, the secretion function of incretin is impaired. Incretin (including GLP-1 and GIP) stimulates insulin secretion and improves insulin sensitivity, which is essential for maintaining glucose homeostasis [3]. Decreased GLP-1 secretion and undermined GIP activity are thought to be involved in the pathogenesis of diabetes [4].

Milk fat globule epidermal growth factor 8 (MFG-E8) is a glycoprotein that was originally found in milk and mammary epithelial cells [5]. In addition to the conventional role of enhancing the clearance of apoptotic cells, increasing evidence suggests that MFG-E8 mediates a multifunctional therapeutic role in reducing inflammation, wound healing, improving prognosis, and also arterial remodeling [6]. In mice with sepsis or dextran sodium sulfate (DSS)-induced colitis, rhMFG-E8 appears to be a benefit in maintaining intestinal mucosal homeostasis and repairing intestinal epithelium [7,8]. Das et al. [9] found that hyperglycemia and exposure to AGEs inactivated MFG-E8, resulting in impaired wound closure in diabetes, while recombinant MFG-E8 resolved inflammation and promoted skin wound healing. It seems that MFG-E8 is beneficial for diabetes-related inflammatory injury, but its mechanisms, especially in the intestine, were not well understood and need to be further explored.

Receptor interacting protein kinase (RIPK) is a key molecule for the programmed death pathway and plays an important role in maintaining a healthy intestinal barrier [10]. Recent studies suggested that, through the release of cellular damage-associated molecular patterns (DAMPs), RIPK1-RIPK3-mixed lineage kinase domain-like (MLKL)-mediated necroptosis could cause many inflammatory diseases, which in turn lead to secondary tissue damage [11]. Thus, inhibitors of the RIPs kinases might be potential therapeutic targets for inflammatory diseases.

In the present study, we detected this necroptosis-related RIPs signaling pathway and established the molecular linkage connecting MFG-E8 with AGEs-stimulated STC-1. We also used D-pinitol, a natural compound that was suggested to have multifunctional properties, such as anti-inflammatory, anti-hyperglycemic, and antioxidant competence, as a protective agent in this model [12]. We provided evidence that the downregulation of MFG-E8 is a meaningful phenomenon in the pathogenesis of diabetes-related intestinal inflammation. MFG-E8 overexpression or D-pinitol treatment notably inhibited the activity of the RIPs pathway and reduced the subsequent release of DAMPs, ultimately improving the secretion function of GLP-1.

## 2. Materials and Methods

### 2.1. Materials

D-pinitol (purity: 95%, Lot No: 441252) and streptozotocin (STZ) were from Sigma Chemicals Co. (St. Louis, MO, USA). Paraformaldehyde (PFA), dimethyl sulfoxide (DMSO), bovine serum albumin (BSA), trypsin/EDTA solution, D-glucose, and 3-(4,5-dimethylthiazol)-2,5-diphenyl-2-H-tetrazolium bromide (MTT) were purchased from Solarbio (Beijing, China). Dulbecco’s Modified Eagle’s Medium (DMEM) and fetal bovine serum (FBS) were obtained from GIBCO (Grand Island, NE, USA). Hoechst 33258 and PI were purchased from Beyotime (Shanghai, China). AGE-BSA was purchased from Abcam (Cambridge, UK). ELISA kits and necrosulfonamide (NSA) were purchased from R&D systems (Minneapolis, MN, USA). Annexin V/PI Kit was from Key Gen (Nanjing, China). The primers were synthesized by Sangon Biotechnology (Shanghai, China). The MFG-E8 antibody was purchased from MBL (Woburn, MA, USA) and R&D (Minneapolis, MN, USA), and antibodies RIPK1, MLKL, phospho-MLKL, and β-action were purchased from Abcam (Cambridge, UK). RIPK3 and HMGB1 antibodies were purchased from Proteintech (Wuhan, China). All other reagents are standard commercial high purity materials.

### 2.2. Animals

Twelve-weeks-old senescence-accelerated mice prone 8 (SAMP8) and senescence-accelerated mice resistant 1 (SAMR1) male mice were all purchased from Peking University Laboratory Animal Centre (Beijing, China). All mice were housed in standard cages with a humidity of 55 ± 5% and a temperature of 20–22 °C and received laboratory pellet chow and tap water ad libitum. The mice were kept under observation for one week prior to the start of the experiment. SAMR1 and SAMP8 mice were randomly divided into the control group (*n* = 10/group) and the diabetes group (*n* = 12/group). The control group was given sham intraperitoneal injections of 0.1mol/L citrate buffer only. The diabetes group received a single intraperitoneal injection of STZ (50 mg/kg) dissolved in freshly prepared 0.1 mol/L sodium citrate buffer (pH 4.5) for five consecutive days. Only mice with fasting blood glucose ≥ 16.7 mmol/L after a week were considered successful in the diabetes model for subsequent experiments. Three mice were excluded from the study as blood glucose was less than 16.7 mmol/L. The study was carried out two weeks after the injection of STZ. None of the mice in each group received any hypoglycemic therapy. All mice fasted overnight and were sacrificed under pentobarbital anesthesia. All the experimental procedures were performed according to the guideline of animal protocol and approved by the Animal Care and Use Committee of Shandong University (Approval No: 18058).

### 2.3. Estimation of Body Weight, Fasting Blood Glucose (FBG)

Mice were weighed weekly. At the end of the experiment, the FBG level was determined with DVI-1650 Automatic Biochemical Analyzer (Bayer, Leverkusen, Germany).

### 2.4. Light Microscopy

The ileum was collected from the distal small intestine and fixed in 4% PFA for 48 h. Then the tissue was embedded in paraffin and cut into 4 μm thick sections. Hematoxylin–eosin (H&E) staining was used for morphological analysis. We measured the villus length from the base to the top of the villi, and the crypt depth from the crypt-villus junction to the crypt base. Immunohistochemistry (IHC) of MFG-E8 was performed according to a standard method. Immunostaining was observed by light microscopy (Nikon Eclipse 80i, Nikon, Tokyo, Japan).

### 2.5. Cell Cultures

STC-1 cells were purchased from the American Type Culture Collection (Rockville, MD, USA). Cells were cultured in DMEM with 10% FBS and 1% penicillin/streptomycin and incubated in a humidified atmosphere containing 5% CO_2_ at 37 °C.

### 2.6. MFG-E8 siRNA and Overexpression Plasmid Transfection

MFG-E8 siRNAs and negative control were designed and chemically synthesized by Suzhou Genepharma Co., Ltd. (Suzhou, China). The siRNA sequence targeting MFG-E8 included: sense 5′- CCAAAUGUCUGGUGACUUUTT-3′, antisense 5′- AAAGUCACCAGACAUUUGGTT-3′. The sequence of negative control siRNA is: sense 5′-GAUGGAGAAGCUCGCUGATTT-3′, antisense 5′- AAAUCAGCGAGCUUCUCCATT’. STC-1 cells were transfected with MFG-E8 siRNA using Lipofectamine 3000 (Invitrogen, Carlsbad, CA, USA) according to the instructions.

MFG-E8 overexpression plasmids were constructed by Suzhou Genepharma Co., Ltd. (Suzhou, China). In simple terms, *mus musculus* MFG-E8 (GenBank accession no: BC003904.1) was cloned into a pcDNA3.1(+) vector. MFG-E8 was amplified by PCR using the forward primer (5′-TCGCAGATCCTTCGCATGCAGGTCTCCCGTGTG-3′) and reverse primer (5′-ATTCTAGAGCTAGCTTAACAGCCCAGCAGCTCCA-3′). The PCR products were double-digested by restriction endonucleases of EcoRI and NotI and then were sub-cloned into the pGFP-C vector. The plasmids with MFG-E8 overexpression or enhanced green fluorescent protein (GFP) were transduced into STC-1 cells with the mediation of lipofectamine 3000 transfection reagent. The expression of MFG-E8 was detected by qPCR and Western blot at 48 h.

### 2.7. Cell Viability Assay

Cell viability was determined by the MTT colorimetric assay following incubation of cells with AGEs (200 ug/mL) with/without pretreatment with D-pinitol (40 uM, 60 uM, 80 uM). STC-1 cells with overexpression plasmids or knockdown of MFG-E8 (5 × 10^4^ cells/mL) were placed into 96-well plates and incubated for 24h and 48 h at 37 °C. Then, 20 μL MTT (5mg/mL) was added to the well and incubated for 4 h. The supernatant was discarded and DMSO was added to solubilize the formazan crystals. The absorbance at 490 nm was determined by an enzyme-labeled analyzer (Thermo Fisher Scientific, Horsham, UK).

### 2.8. Cell Death Assay

Cell death was assessed by flow cytometry, and PI and Hoechst staining. Following incubation with AGEs (200 ug/mL) with/without pretreatment with D-pinitol (40 uM, 60 uM, 80 uM), cells were centrifuged and washed with ice-cold PBS. Then, cells were resuspended in 100 μL binding buffer containing 5 μL Annexin V/FITC and 5 μL PI. Afterwards, cells were incubated in the dark for 15 min at room temperature and detected by flow cytometry (Beckman CytoFlex, Pasadena, CA, USA) using ModFit software within 30 min. Annexin V(+)/PI(+) cells in the upper right quadrant were considered necrotic.

The cultured cells were stained with Hoechst 33258 (5 μL/mL) at 4 °C for 10 min and PI (5 μL/mL) at 37 °C for 15 min. The cells were then fixed with 4% PFA for 15 min at room temperature. Stained samples were analyzed by fluorescence microscope (Olympus IX73, Tokyo, Japan).

### 2.9. ELISA Assay

The content of HMGB1, TNF-α, IL-6, IL-1β, and GLP-1 in culture supernatants was detected with ELISA kits. The concentration was calculated from a standard curve generated simultaneously. Results are presented as pg/mL or ng/mL.

### 2.10. Quantitative Real-Time PCR

After STC-1 cells harvesting, total RNA was extracted and reverse transcribed into cDNA with the SureScript™ First-Strand cDNA Synthesis Kit (GeneCopoeia, Rockville, MD, USA). Quantitative analysis of the MFG-E8 mRNA was performed by the RT-PCR method using the BlazeTaq™ SYBR Green qPCR Mix 2.0 (GeneCopoeia, Rockville, MD, USA). GAPDH mRNA was used as an internal control. The primer sets sequences were as follows: MFGE8 (forward) 5′-CTCTGGAGGCACAGTACATAAAG-3′, (reverse) 5′-TCAGAACATCCGTGCAACTC-3′; GAPDH (forward) 5′-GAGGTATCCTGACCCTGAAGTA-3′, (reverse) 5′-CACACGCAGCTCATTGTAGA-3′. All samples were detected in triplicate.

### 2.11. Western Blot Assay

The total protein of intestine tissue and cells was extracted. An equal amount of protein was separated by sodium dodecyl sulfate polyacrylamide-gel electrophoresis (SDS-PAGE) and was then transferred onto the polyvinylidene fluoride membranes (PVDF). The membranes were incubated in blocking solution containing 5% nonfat dry milk for 1 h at room temperature, and then incubated overnight at 4 °C with antibodies for MFG-E8, RIPK1, RIPK3, MLKL, and phosphorylated MLKL (p-MLKL). The membranes were washed with TBS-T, incubated with HRP-conjugated secondary antibody, and visualized using an enhanced chemiluminescence kit (Thermo Pierce). β-actin was used as a loading control to ensure the equal loading of protein.

### 2.12. Immunocytochemical Staining

After the cells were processed, the slides were fixed and incubated overnight with the primary antibody against p-MLKL at 4 °C. Next, the slides were incubated with a secondary antibody with biotin-labeled (Jackson immunoresearch) for 1 h and goat-horseradish peroxidase polymer for another 1 h. Then, the slides were added with DAB to stain the cell nuclei and visualized using a light microscope (Nikon Eclipse 80i, Nikon, Tokyo, Japan).

### 2.13. Statistical Analysis

Data were presented as the means ± standard deviation (SD). Results were analyzed using Student’s *t*-tests or one-way analysis of variance (ANOVA). GraphPad Prism 8 software package (GraphPad Software, San Diego, CA, USA) was used to perform the statistical analysis and graphics. *p* < 0.05 was considered statistically significant.

## 3. Results

### 3.1. Body Weight and FBG in Control and Diabetic Mice

**Figure 1:**
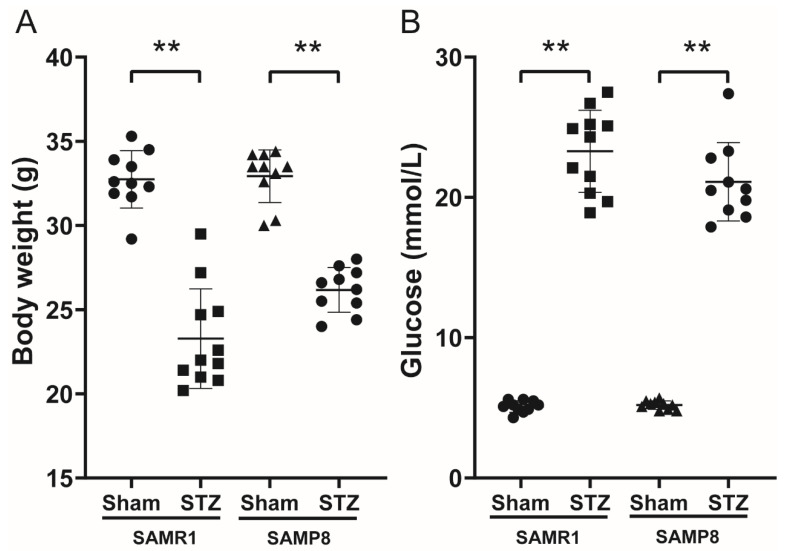
Body weight and FBG in control and diabetic mice. (**A**) Body weight changes of the mice for two weeks after diabetic model established (*n* = 10–11 per group). (**B**) FBG levels of the mice for two weeks after diabetic model established (*n* = 10–11 per group). ** *p* < 0.01 vs. SAMR1/SAMP8 control group. FBG: fasting blood glucose.

After two weeks, the body weights and FBG were compared in SAMR1/SAMP8 control mice and STZ-induced diabetic mice. There were statistically significant hyperglycemic levels and a slight reduction in body weights in both sets of STZ-treated mice (Figure 1A,B).

### 3.2. Histological Findings and the Expression of MFG-E8, p-MLKL and HMGB1 in the Intestinal Tissues

**Figure 2:**
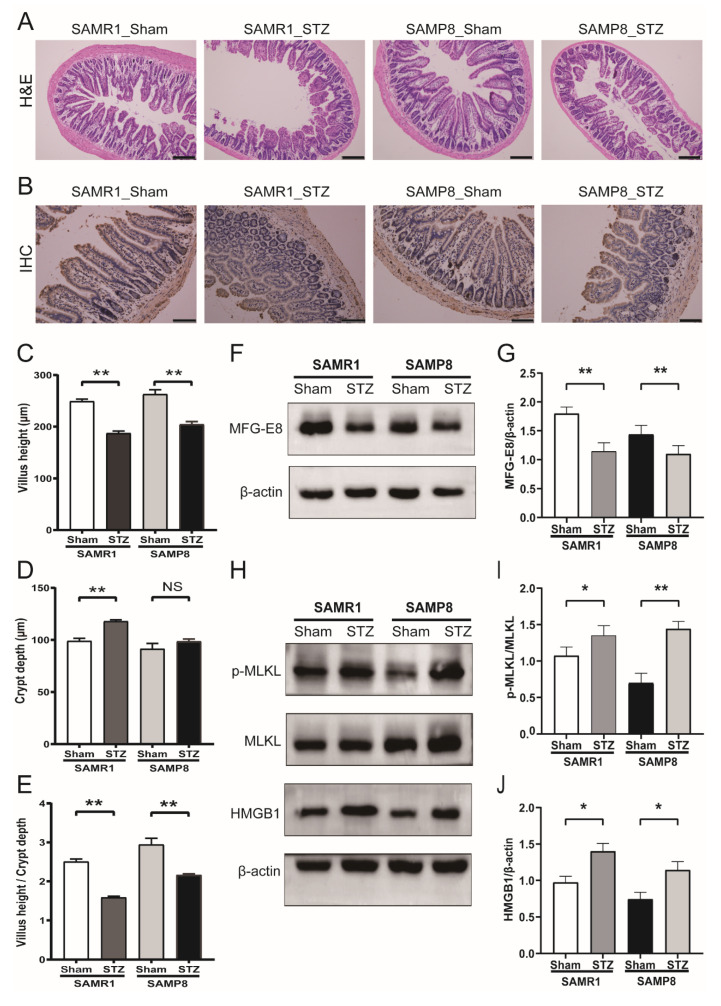
Histological findings and the protein expression of MFG-E8, p-MLKL and HMGB1 in the intestinal. (**A**) Representative H&E staining of terminal ileum section was assayed (scale bar: 200 um, *n* = 5 per group). (**B**) Immunohistochemical staining for protein localization of MFG-E8 (scale bar: 100 um, *n* = 5 per group). (**C**–**E**) Intestinal villus height, crypt depth, and the ratio between the two were measured in each group to evaluate the intestinal morphology (*n* = 5 per group). (**F**–**J**) Western blot and densitometric analyses of MFG-E8, p-MLKL, and HMGB1 protein expression in the small intestine of the four groups. * *p* < 0.05, ** *p* < 0.01 vs. SAMR1/SAMP8 control group. NS: no statistical difference.

H&E staining showed that the morphology of the ileum rapidly changed two weeks later after STZ was administered (Figure 2A). In several parts, the sloughing of intestinal epithelial was occasionally seen. The villus became blunt and shorter, and the villus height/crypt depth ratio evidently decreased in the diabetes group compared with that in the control group (Figure 2C–E). We then performed immunohistochemistry to explore the localization and expression of MFG-E8. The positive cells appeared brown in color and were mostly located on the surface of the intestinal villus and crypts of normal intestinal tissues (Figure 2B). Compared with controls, diabetic mice demonstrated a significant decrease in MFG-E8-positive rate, with expression mainly reduced in the base of villus and crypts.

To further verify the change in MFG-E8 protein expression during the process, we used the Western blotting analysis. The result showed that the protein expression of MFG-E8 was abundant in intestinal tissues of SAMR1 and SAMP8 mice, while the expression of MFG-E8 in two groups of STZ-induced diabetic mice was significantly reduced in a similar trend (Figure 2F,G, *p* < 0.01). The result suggested that diabetes reduced the expression of MFG-E8 in the SAMR1 mice but did not further decrease it in the SAMP8 mice where it was already significantly reduced. Since diabetic mice exhibited marked impairment in the intestinal epithelium, we measured the expression of p-MLKL and HMGB1 to determine whether necroptosis-related hallmarks were affected in vivo. We found that elevated p-MLKL and HMGB1 expression were detected in diabetic mice (Figure 2H–J, *p* < 0.05). These results indicated that MFG-E8 might be involved in diabetes-related intestinal epithelium damage and that necroptosis might be a potential regulatory mechanism during the process.

### 3.3. MFG-E8 siRNA and Overexpression Plasmids Transduction Efficiency

**Figure 3:**
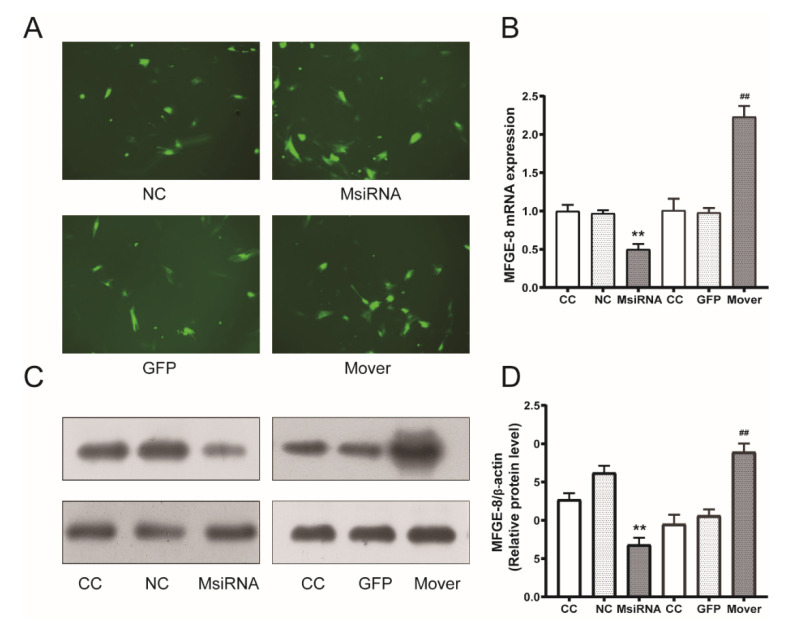
MFG-E8 small interfering RNA (siRNA) and overexpression plasmid transfection of STC-1. (**A**) Fluorescence micrograph displays MFG-E8 siRNA and overexpression in STC-1 cells at 48 h after transfection (×200). (**B**) Quantitative RT-PCR analysis demonstrates MFG-E8 mRNA expression in STC-1 at 48 h after transfection. (**C**) Western blot analysis demonstrates MFG-E8 protein expression in STC-1 at 48 h after transfection. (**D**) Data were expressed as the expression ratio of MFG-E8/β-actin. ** *p* < 0.01 compared with NC group; ^##^ *p* < 0.01 compared with GFP group. CC group: normal control cells; NC group: negative control siRNA cells; MsiRNA group: STC-1 carrying siRNA against MFG-E8; GFP group: STC-1 carrying GFP; Mover group: STC-1 carrying both GFP and MFG-E8.

STC-1 cells were transduced with siRNA or overexpression plasmids. The transduction efficiency was assessed by fluorescence microscopy, qPCR, and Western blotting. STC-1 carrying negative control siRNA (NC), MFG-E8 siRNA (MsiRNA), STC-1 carrying EGFP (GFP), both EGFP and MFG-E8 genes (Mover) were harvested. Both siRNA and overexpression plasmids reached optimal transduction efficiency at 48 h (Figure 3A–D, *p* < 0.01).

### 3.4. Effects of AGEs, D-Pinitol, MFG-E8 siRNA and Overexpression Plasmids on the STC-1 Cell Viability

**Figure 4:**
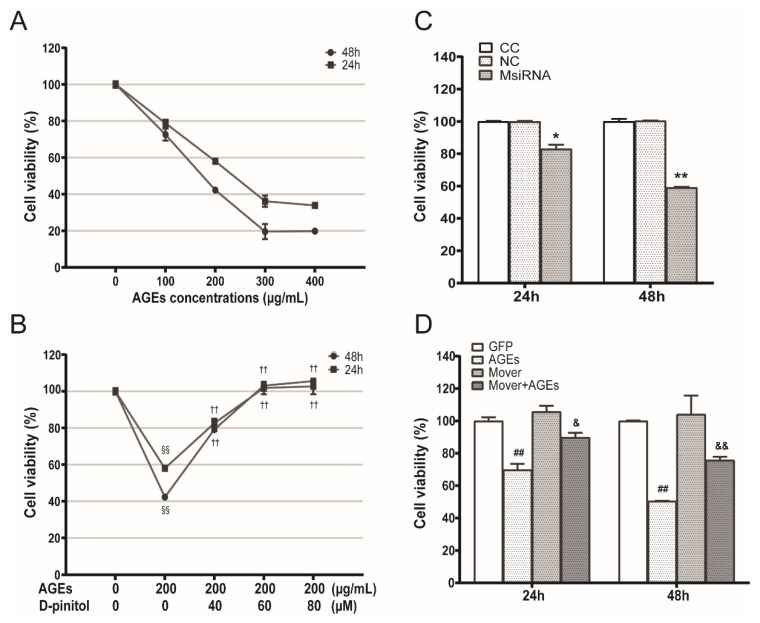
Role of MFG-E8 and D-pinitol on the cell viability of STC-1 treated with AGEs. (**A**) Effects of different concentrations of AGEs (0, 100, 200, 300, 400 μg/mL) on viability in STC-1 at 24 h and 48 h with MTT. (**B**) Effects of D-pinitol on viability in STC-1 treated by AGEs at 24 h and 48 h with MTT. (**C**) Effects of MFG-E8 siRNA on viability in STC-1 at 24 h and 48 h. (**D**) Effects of MFG-E8 overexpression on viability in STC-1 treated by AGEs. Results were expressed as percent of untreated cells (100%) and are given as the means ± SD from six independent experiments. ^§§^ *p* < 0.01 vs. CC group; ^††^ *p* < 0.01 vs. CC + AGEs group; * *p* < 0.05, ** *p* < 0.01 vs. NC group; ^##^ *p* < 0.01 vs. GFP group; ^&^ *p* < 0.05, ^&&^ *p* < 0.01 vs. GFP+AGEs group. AGEs: advanced glycation end products.

To detect the effect of AGEs on the viability of STC-1 cells and detect the protective effect of D-pinitol, we used an MTT assay. We found that the cell viability was decreased in a dose-dependent manner when STC-1 was exposed to AGEs (0, 100, 200, 300, 400 ug/mL) for 24 h and 48 h. The AGEs concentration (200 ug/mL), which decreased ~60% of the cell viability, was chosen in all later experiments (Figure 4A). Different concentrations of D-pinitol (40, 60, 80 uM) dose-dependently increased AGEs-induced decrease in cell viability (Figure 4B).

To further study the effect of MFG-E8 on STC-1 viability, we transfected STC-1 cells with MFG-E8 siRNA and overexpression plasmids and estimated cell viability. NC or GFP did not significantly affect cell viability. We found that MFG-E8 siRNA caused significant a decrease in STC-1 cell viability compared with the NC group at 24 h and 48 h (Figure 4C, *p* < 0.05), whereas overexpression of MFG-E8 attenuated AGEs-induced cell viability decrease at 24 h and 48 h (Figure 4D, *p* < 0.05).

### 3.5. Role of D-Pinitol on AGEs-Induced Necroptosis and Effects of MFG-E8 siRNA and Overexpression Plasmids

**Figure 5:**
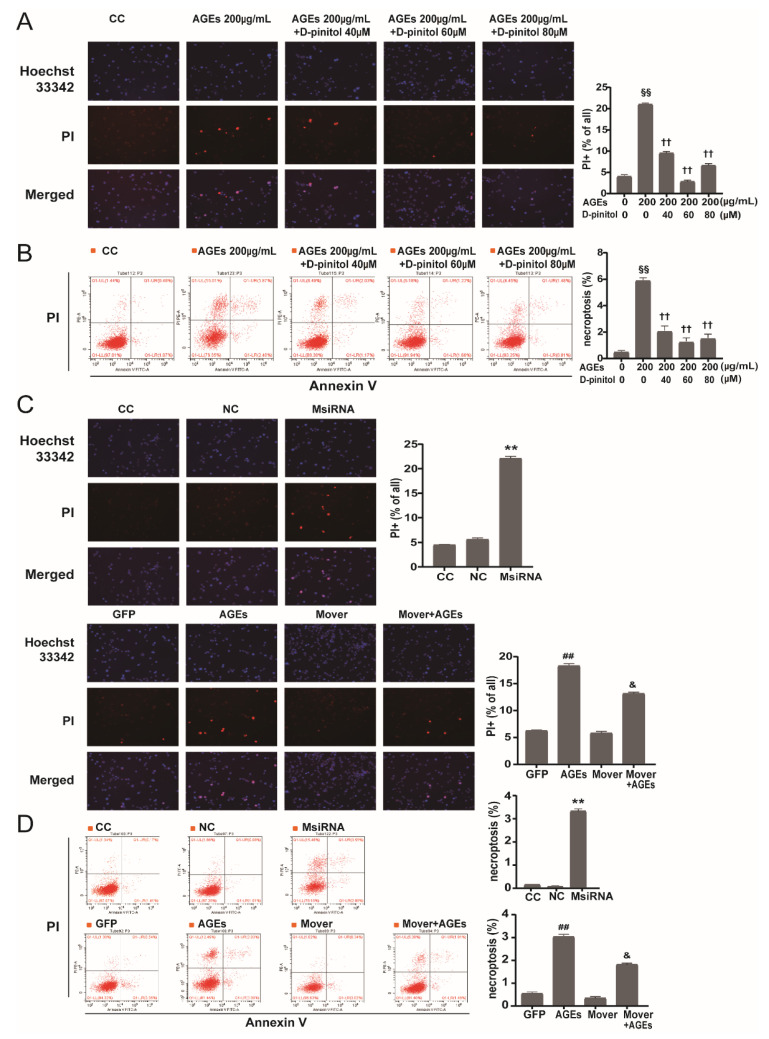
Role of MFG-E8 and D-pinitol on AGEs-induced necroptosis in STC-1 cells. (**A**) Representative images of PI (red) and Hoechst (blue) of STC-1 subjected to AGEs stimulation that with/without different concentrations of D-pinitol pretreatment. (**B**) Representative flow cytometry images of STC-1 stained with PI/Annexin V from three independent experiments. The necroptosis activation is represented by ratio of PI+/Annexin V+. ^§§^ *p* < 0.01 vs. CC group; ^††^ *p* < 0.01 vs. CC+AGEs group. (**C**) Representative images of PI (red) and Hoechst (blue) of STC-1 subjected to MFG-E8 siRNA and overexpression. (**D**) Representative flow cytometry images of STC-1 stained with PI/Annexin V (Bar: 50 µm, *n* = 3). The necroptosis activation was represented by ratio of PI (+)/Annexin V (+). ** *p* < 0.01 vs. NC group; ^##^ *p* < 0.01 vs. GFP group; ^&^ *p* < 0.05 vs. GFP+AGEs group. AGEs: advanced glycation end products.

PI labeling was used to determine whether AGEs could induce STC-1 cells death. We chose 24 h time point for the measurements. As shown in Figure 5A, AGEs (200 ug/mL) caused a pronounced increase in PI-positive cells. Flow cytometry results in Figure 5B revealed that D-pinitol pretreatment caused a decrease in PI-positive cells (red fluorescent that indicating dead cells) in a dose-dependent manner. In addition, the results of Annexin V/PI flow cytometry showed a similar trend to that of the PI/Hoechst staining analysis, in which the morphological characteristics of typical necroptosis were inhibited by co-cultured with D-pinitol. When the concentration of D-pinitol increased to 80 μM, the protective effect became weak and PI (+)/Annexin V(+) cells slightly increased.

We next determined whether MFG-E8 is involved in the regulation of necroptosis by transfecting STC-1 cells with MFG-E8 siRNA or overexpression. As the PI/Hoechst staining showed, the number of PI+ cells was significantly increased in the MFG-E8 siRNA group (Figure 5C), which indicated that the endothelial necroptosis was activated after MFG-E8 silencing. In addition, necroptosis also occurred in the AGEs-treated group, and MFG-E8 overexpression attenuated AGEs-induced necroptosis. The results of Annexin V/PI flow cytometry were similar to that of PI/Hoechst staining (Figure 5D).

### 3.6. Effects of D-Pinitol on MFG-E8 and Pro-Inflammatory Cytokines in AGEs Treated STC-1 Cells

**Figure 6:**
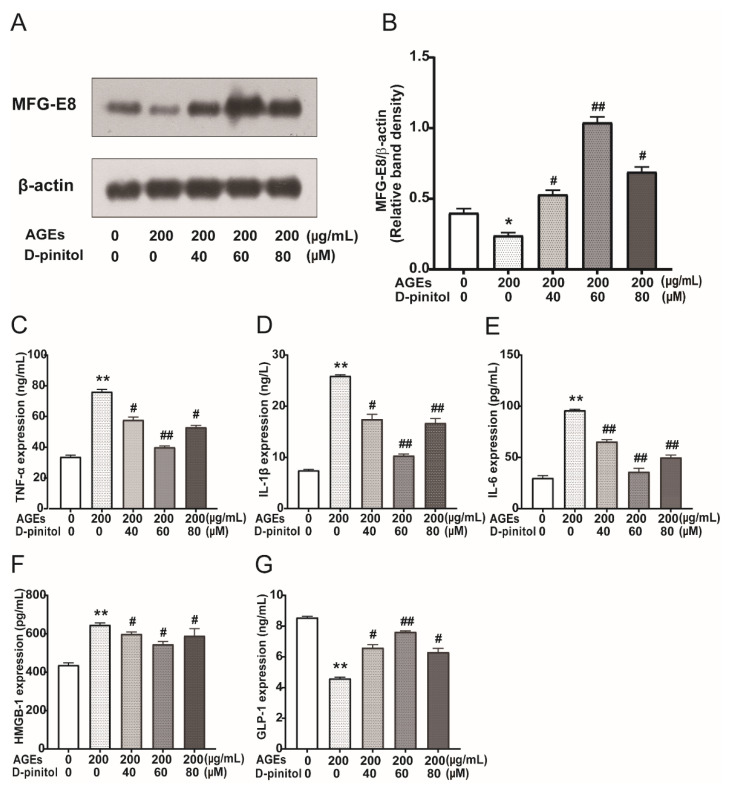
Effects of D-pinitol on MFG-E8 and pro-inflammatory cytokines. (**A**) Western blot showing effects of D-pinitol on the expression of MFG-E8 protein levels in STC-1 exposed to AGEs. (**B**) Data were expressed as the expression ratio of MFG-E8/β-actin. (**C**–**G**) ELISA assay revealing HMGB1, TNF-α, IL-1β, IL-6, and GLP-1 release in presence of AGEs with or without D-pinitol. Results were expressed as the means ± SD from three independent experiments. * *p* < 0.05, ** *p* < 0.01 vs. CC group; ^#^ *p* < 0.05, ^##^ *p* < 0.01 vs. CC + AGEs group. AGEs: advanced glycation end products.

The protein expression of MFG-E8 was significantly decreased after AGEs stimulation, and D-pinitol pre-treatment led to the upregulation of MFG-E8 compared with that in the normal control group and AGEs-treated group (Figure 6A,B, *p* < 0.05). Massive DAMPs released from the necroptotic cells could trigger an inflammatory response. We were interested in exploring whether the increase in AGEs-induced necroptosis was with the release of DAMPs. As shown in Figure 6C–F, HMGB1, as a critical cytokine of DAMPs, was significantly increased in AGEs-treated STC-1 cells, accompanied by the increase in pro-inflammatory cytokines TNF-α, IL-1β, and IL-6, while D-pinitol pro-treatment decreased these levels (*p* < 0.05). We examined whether AGEs treatment affected GLP-1 expression in STC-1 and found that AGEs treatment could inhibit GLP-1 levels in cell supernatant, while D-pinitol pre-treatment could partially increase the GLP-1 levels (Figure 6G).

### 3.7. Effect of MFG-E8 on the Expression of DAMPs and Pro-Inflammatory Cytokines in STC-1 Cells

**Figure 7:**
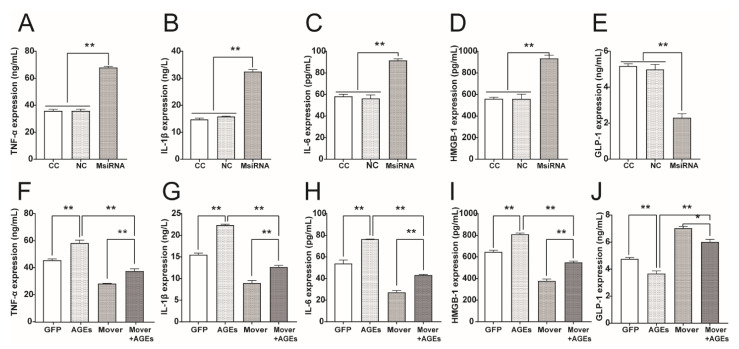
ELISA assay revealing effect of MFG-E8 on DAMPs and pro-inflammatory cytokines in STC-1. (**A**–**E**) Effects of MFG-E8 siRNA on HMGB1, TNF-α, IL-1β, IL-6, and GLP-1 levels in STC-1. (**F**–**J**) Effects of MFG-E8 overexpression on HMGB1, TNF-α, IL-1β, IL-6, and GLP-1 levels in STC-1. Results were expressed as the means ± SD from three independent experiments. * *p* < 0.05, ** *p* < 0.01. DAMPs: damage-associated molecular patterns.

Reducing DAMPs including HMGB1 release could subsequently reduce the pro-inflammatory response. We wondered if changes in MFG-E8 could affect inflammatory factors. MFG-E8 siRNA increased HMGB1 and the pro-inflammatory cytokines of TNF-α, IL-1β, IL-6, and led to a decrease in GLP-1 levels (Figure 7A–E, *p* < 0.01). On the other hand, MFG-E8 overexpression attenuated AGEs-induced upregulation of HMGB1 and pro-inflammatory cytokines in STC-1 cells, resulting in a decrease in the inflammatory cytokines and an increase in GLP-1 levels (Figure 7F–J, *p* < 0.01).

### 3.8. NSA Reversed AGEs-Induced Phosphorylation of MLKL and Upregulation of Pro-Inflammatory Cytokines

**Figure 8:**
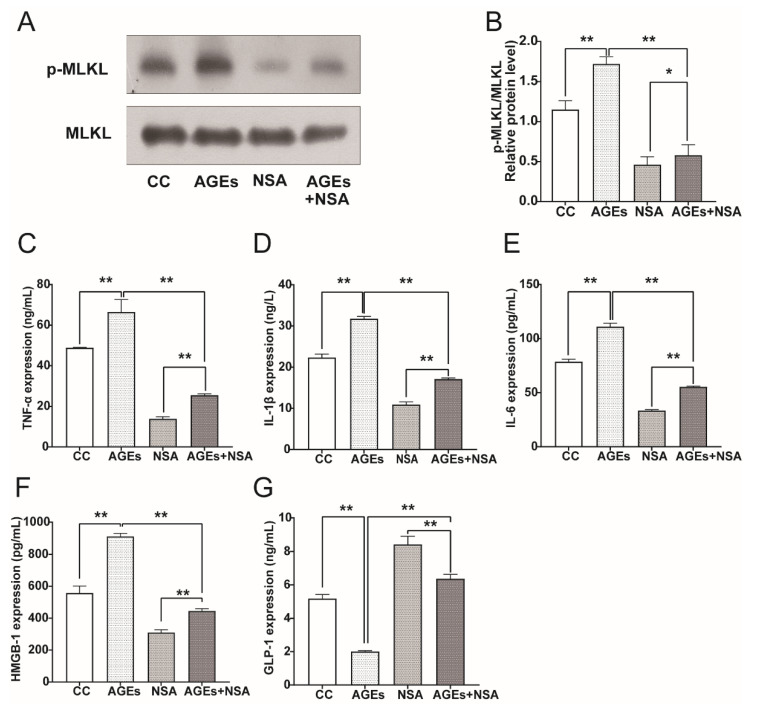
NSA partially reversed AGEs-induced phosphorylation of MLKL and upregulation of pro-inflammatory cytokines. (**A**) Western blot showing p-MLKL protein level in STC-1 cells in presence of AGEs with or without MLKL inhibitor NSA. (**B**) The histograms represent the means of three experiments ± SD. (**C**–**G**) ELISA assay revealing HMGB1, TNF-α, IL-1β, IL-6, and GLP-1 release in STC-1 cells in presence of AGEs with or without NSA. Data were expressed as the means ± SD from three independent experiments. * *p* < 0.05, ** *p* < 0.01. NSA: necrosulfonamide.

As MLKL is a key effector molecule that executes necroptosis, and its activity enhances systematic inflammation, we used MLKL-specific inhibitor NSA to block the necroptosis pathway and detect the expression of p-MLKL and pro-inflammatory cytokines in STC-1 cells. We found a 1.5-fold increase in levels of p-MLKL protein expression after AGEs treatment, whereas NSA pretreatment significantly attenuated AGEs-induced activation of p-MLKL (Figure 8A,B, *p* < 0.05).

ELISA analysis showed that, after being stimulated with AGEs, HMGB1 was released from STC-1 cells, resulting in increased expression of pro-inflammatory cytokines such as TNF-α, IL-1β, and IL-6. However, NSA pretreatment almost completely terminated HMGB1 production through inhibition of MLKL function, as with the reduction in pro-inflammatory cytokines TNF-α, IL-1β, and IL-6. (Figure 8C–F, *p* < 0.01). The secretion of GLP-1 in the supernatant was opposite to that of pro-inflammatory factors (Figure 8G, *p* < 0.01). This suggested that inhibition of p-MLKL signaling could achieve affirmative anti-inflammatory effects.

### 3.9. Effect of MFG-E8 on Necroptosis Pathway Related Protein Expression in STC-1 Cells

**Figure 9:**
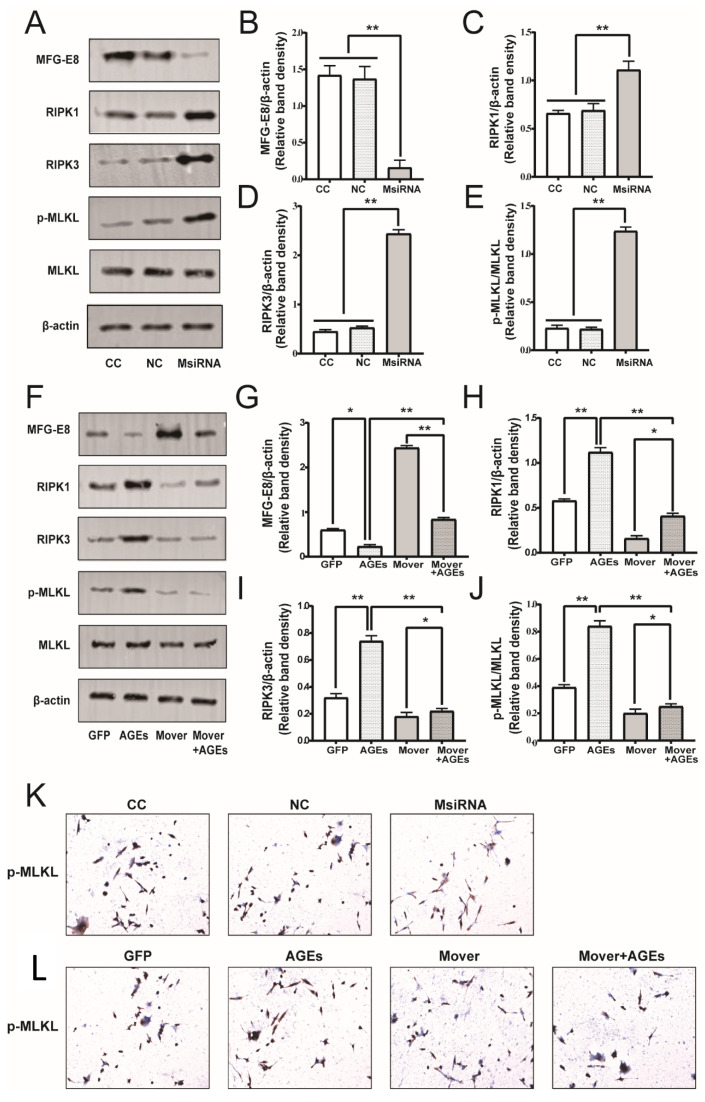
Effects of MFG-E8 on the necroptosis pathway related protein expression in STC-1 cells. (**A**) Effects of MFG-E8 siRNA on the expression of RIPK1-RIPK3-MLKL pathway. (**B**–**E**) Data were expressed as the expression ratio of MFG-E8/β-actin, RIPK1/β-actin, RIPK3/β-actin, p-MLKL/MLKL. (**F**) Effects of MFG-E8 overexpression on the expression of RIPK1-RIPK3-MLKL pathway. (**G**–**J**) Data were expressed as the expression ratio of MFG-E8/β-actin, RIPK1/β-actin, RIPK3/β-actin, p-MLKL/MLKL. (**K**,**L**): ICC staining showed the protein level of p-MLKL-positive cells in MFG-E8 siRNA and overexpression group. Data were expressed as the means ± SD from three independent experiments. * *p* < 0.05, ** *p* < 0.01.

To further explore the molecular mechanism of MFG-E8 in pro-inflammatory STC-1, we examined the expression of the RIP1–RIP3–MLKL pathway, which is crucial for the induction of cytokines related to necroptosis. The RIPK1, RIPK3, and p-MLKL levels were significantly increased in the MFG-E8 siRNA group compared to that in the NC group (Figure 9A–E, *p* < 0.05). Stimulation of the STC-1 cells with AGEs resulted in a significant increase in RIPK1, RIPK3, and p-MLKL expression, while overexpression of MFG-E8 attenuated the activation of the RIPs pathway induced by AGEs (Figure 9F–J, *p* < 0.05).

Consistent with the Western blot results, the ICC staining showed that p-MLKL-positive cells were significantly higher in the siMFG-E8 and AGEs-treated group (Figure 9K). On the other hand, MFG-E8 overexpression diminished AGEs-induced activation of p-MLKL in STC-1 cells (Figure 9L). The results indicate that MFG-E8 may regulate the inflammatory response through the RIPs pathway and then regulate the release of GLP-1 in STC-1 cells.

## 4. Discussion

Longstanding hyperglycemia leads to potentially destructive biochemical and physiological consequences throughout the body through the accumulation of AGEs [13]. During this process, dysfunction of enteroendocrine L-cell is mainly due to the impairment of the intestinal epithelial barrier and has been reported for the reduction in GLP-1 secretion [14]. This suggests that AGEs may be involved in the regulation of incretin secretion, although the underlying molecular mechanism remains unclear.

In the present in vivo study, we found that STZ treatment could aggravate the pathological change in the small intestine, which means that high glucose toxicity can promote intestinal epithelial injury and in turn affects its absorption and enteroendocrine function. Moreover, diabetic mice presented a marked decrease in MFG-E8 expression, accompanied by higher p-MLKL and HMGB1 expression in the ileum section, which indicated that necroptosis-related inflammatory reaction mainly occurs in the intestinal epithelial and MFG-E8 might play a role in the process. In an in vitro study of an AGEs-induced intestinal injury model, we observed the activity of necroptosis-related inflammation and its negative effect on the secretory function of intestinal endocrine cell STC-1. Overexpression of MFG-E8 attenuated AGEs-induced inflammatory necrosis in STC-1 cells and reduced the activity of the RIPs pathway in the model. Our study provides evidence for the relationship between AGEs, MFG-E8, and intestinal inflammation. The results indicate that the downregulation of MFG-E8 is likely to be involved in the mechanism of diabetes-related intestinal inflammation and the reduction in inflammatory response caused by the upregulation of MFG-E8 may be partly due to the inhibition of the RIPs pathway.

MFG-E8 is a peripheral glycoprotein that exhibits broad functions in a variety of diseases, such as sepsis, cancers, cardiac ischemia-reperfusion injury, and vascular aging-related diseases [9,15,16]. In addition, previous investigations have found that MFG-E8 plays an important role in the repair of the intestinal barrier and intestinal mucosa, but the specific mechanism is not yet clear [7]. Studies have proved that the loss or inactivation of MFG-E8 is closely related to the decline of TNF-α, IL-6, IL-1β, and other pro-inflammatory factors in the process of inflammation, necrosis, and oxidative stress. Recombinant rhMFG-E8 can inhibit the release of inflammatory factors in injury models [17,18]. A recent study found that MFG-E8 can promote angiogenesis and wound closure in diabetic patients by regulating the “NLRP3 inflammasome”, and is closely related to representative molecules DAMPs such as HMGB1 [19]. We speculate that MFG-E8 may be related to necrotic related inflammation.

We treated STC-1 cells with AGEs to induce a necroptosis-related inflammation model. We observed a decrease in MFG-E8 expression accompanied by an increase in necrotic cells and inflammatory factors. D-pinitol pretreatment had a protective effect. D-pinitol as a natural compound was found to have anti-inflammatory [20], antioxidant, and even anti-hyperglycemic effects [21]. The results suggest that there may be negative regulation between MFG-E8 and intestinal inflammation, and anti-inflammatory treatment may promote the upregulation of MFG-E8.

Programmed cell death plays an important role in the lifelong maintenance of tissue homeostasis, especially intestinal epithelial homeostasis and mucosal barrier protection [22,23]. Necroptosis is a regulated, protease-independent cell death that involves the activation of specific mediators such as RIPK1, RIPK3, and MLKL. Despite the RIPs axis having been widely investigated in cell death and inflammatory signaling, the viewpoint that RIPs predominantly drive inflammation through necroptosis-independent mechanisms has recently gained much attention [24]. In animal models of acute pancreatitis, ischemic injury [25], and neurodegeneration [26] that are related to RIPK1/RIPK3 deficiency or MLKL knockout, it has been suggested that necroptosis may be a key element in triggering inflammation [27]. However, little is known about the function of necroptosis in intestinal inflammation and whether its inhibition can serve as a therapeutic strategy. In the intestinal epithelial of FADD-deficient mouse, both epithelial cell death and inflammation in the mouse colon and small intestine were prevented by RIPK3 deficiency, providing evidence for RIPK3-mediated epithelial cell necroptosis and intestinal inflammation [28].

Hence, we investigated inflammation and necroptosis in vitro, following AGEs-induced cell injury, by analyzing the expression of the RIPs and alteration of cytokines. We showed that AGEs treatment activated the RIP3-pMLKL pathway and remarkably increased the production of inflammatory molecules, such as TNF-α, IL-1β, IL-6, and the HMGB1 acting as a DAMP/alarmin. It indicates that AGEs drive necroptosis-induced inflammation. Recent insights into the mechanism of necroptosis have shown that RIP3-mediated phosphorylation could activate cytoplasmic MLKL. P-MLKL as an executor that ultimately induces necroptosis, causing membrane rupture and DAMPs release [29]. In our study, we observed activation of p-MLKL accompanied by the release of pro-inflammatory mediators, while after treatment with the MLKL inhibitor (NSA), the release of downstream pro-inflammatory mediators decreased. A recent in vitro and ex vivo study showed that RIPK3-induced necroptosis alters epithelial permeability by triggering MLKL activation and increasing cytokine/alarmin expression (IL-1β, IL-8, IL-33, and HMGB1) [30]. Thus, activation of p-MLKL predominantly drives inflammation through necroptosis-related mechanisms, and inhibition of necroptosis may be of therapeutic importance to reduce intestinal inflammation and needs further investigation.

To explore the specific mechanism of MFG-E8 in the necrotizing inflammatory response, we observed the changes in the RIPs pathway by intervening MFG-E8. The results showed that the RIPs pathway could be activated after MFG-E8 silencing, accompanied by the increase in inflammatory factors. In addition, MFG-E8 overexpression attenuated the activity of the RIPs pathway and inhibited the inflammatory factor high expression stimulated by AGEs. Therefore, we reasoned that MFG-E8 might alter inflammatory factor expression through the RIPs pathway and in turn affect GLP-1 secretion in STC-1 cells.

In conclusion, we provided evidence that AGEs could induce necro-inflammation in STC-1 cells accompanied by the downregulation of MFG-E8, during which the inflammatory response is promoted through necroptosis-related mechanisms. Overexpression of MFG-E8 or exogenous D-pinitol treatment can reduce the necro-inflammation in STC-1 cells and ultimately improve the GLP-1 secretion function. Our findings support a meaningful role for MFG-E8 in necroptosis-induced intestinal inflammatory responses in diabetes and indicate MFG-E8 as a potential targeted therapeutic strategy for maintaining glucose homeostasis.

## Data Availability

All data in this study are available if requested.
